# Global climatological data of ocean thermohaline parameters derived from WOA18

**DOI:** 10.1038/s41597-023-02308-7

**Published:** 2023-06-24

**Authors:** Peter C. Chu, Chenwu Fan

**Affiliations:** Department of Oceanography, Naval Postgraduate School, Monterey, CA 93943 USA

**Keywords:** Physical oceanography, Fluid dynamics

## Abstract

This is a global ocean climatological dataset of 17 thermohaline parameters such as isothermal layer (ITL) depth (*h*_*T*_), mixed layer (ML) depth (*h*_*D*_), thermocline gradient (*G*_*T*_), pycnocline gradient (*G*_*D*_) determined from temperature (*T*) and salinity (*S*) profiles of the National Centers for Environmental Information (NCEI) world ocean atlas 2018 (WOA18) using the double gradient method along with the identity-index (*i*-index) showing the quality of the determination. With the identified (*h*_*T*_, *h*_*D*_) and (*G*_*T*_, *G*_*D*_), other parameters such as ITL heat content (*H*_*ITL*_), mixed layer fresh-water content (*F*_*ML*_), maximum thermocline gradient (*G*_*Tmax*_), thermocline depth (*h*_*th*_), temperature at thermocline depth (*T*_*th*_), maximum density gradient *G*_*Dmax*_), pycnocline depth (*h*_*pyc*_), density at pycnocline depth (*ρ*_*pyc*_), and (*h*_*T*_ − *h*_*D*_) (barrier layer if positive or compensated layer if negative). The dataset is located at the NOAA/NCEI website (10.25921/j3v2-jy50). It provides useful background information for ocean mixed layer dynamics, air-sea interaction, climatological studies. This paper is the only document for the dataset.

## Background & Summary

Ocean has upmost layer with near-zero vertical gradient such as isothermal layer (ITL) for temperature (*T*), or mixed layer (ML) for density (ρ) and underneath layer with strong vertical gradient such as thermocline (for *T*), or pycnocline (for *ρ*)^1^. Temperature and salinity (S) are observed in oceanography. Usually, the Thermodynamic Equation of Seawater-2010 (https://www.teos-10.org/) is used to compute *ρ* from (*T*, *S*) data. Thermocline or pycnocline with strong vertical gradient is the transition layer between the vertically quasi-uniform layer from the surface (ITL or ML), and the deep-water layer. The vertically quasi-uniform ITL and ML are caused by intense turbulent mixing near the ocean surface. Such mixing is driven by shear due to surface wind stress and by convection due to heat loss from ocean to atmosphere. The thermocline (pycnocline) resists the turbulent mixing from the ITL (or ML) and limits the ITL (or ML) deepening and heat (or water mass) exchange between the ITL (or ML) and deeper layer due to its strong vertical gradient. The thermocline gradient *G*_*T*_ (or pycnocline gradient *G*_*D*_) directly affects such exchange^2–4^.

**Fig. 1:**
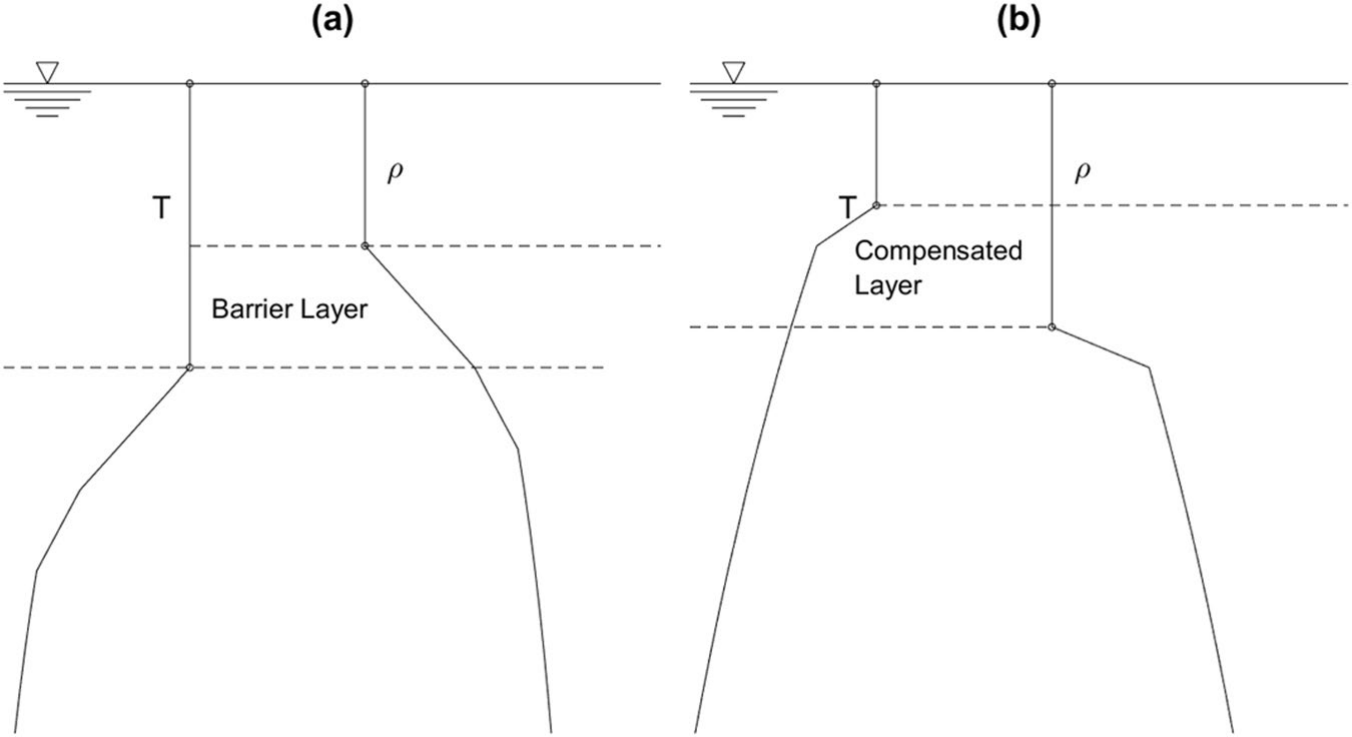
Schematic illustration of (T, *ρ*) profiles for occurrence of (**a**) barrier layer when ILD > MLD, and (**b**) compensated layer when ILD < MLD.

The ITL and ML provide dynamic-thermodynamic links and mediates the exchange of momentum, heat, and moisture between the atmosphere and the oceans; and hence plays a key role to affect weather and climate. Such exchange depends on an important parameter, i.e., the ocean isothermal layer depth (ILD) *h*_*T*_ [or mixed layer depth (MLD) *h*_*D*_)], which determines the heat content (or freshwater content) and mechanical inertia of the layer. Temporal variability of *h*_*T*_ (or *h*_*D*_) is caused by many processes occurring in the isothermal (mixed) layer such as surface forcing, lateral advection, internal waves, etc., ranging from diurnal, seasonal, to interannual variability^2–4^. Spatial variability of *h*_*T*_ (or *h*_*D*_) is evident from less than 20 m in summer to more than 500 m in winter in subpolar latitudes^5^. Therefore, determination of the four parameters (*h*_*T*_, *h*_*D*_, *G*_*T*_, *G*_*D*_) from (*T*, *ρ*) profiles becomes important. Note that the two depths (*h*_*T*_, *h*_*D*_) are not necessary the same with the occurrence of barrier layer if (*h*_*T*_ > *h*_*D*_)^6–15^ (Fig. 1a), and compensated layer if (*h*_*T*_ < *h*_*D*_)^15^.

Let a *T*-profile (or ρ-profile) starting from the ocean surface down to depth *z*_*k*_ be represented by *T*(*z*_*k*_) [or *ρ*(*z*_*k*_)]. *k* = 1, 2, …, *K*, with *z*_1_ the surface and *z*_*K*_ the bottom of the profile. The corresponding vertical gradient is represented by *G*_*T*_(*z*_*k*_) [or *G*_*D*_(*z*_*k*_)]. The vertical gradient for upper ocean ITL-thermocline (or ML-pycnocline) is depicted by

1 $$\left\{\begin{array}{l}{G}_{T}({z}_{k})\approx 0\;{\rm{for}}\;{z}_{k} > -\,{h}_{T}\;{\rm{(in}}\;{\rm{ITL),}}\quad \quad \quad {G}_{D}({z}_{k})\approx 0\;{\rm{for}}\;{z}_{k} > -\,{h}_{D}\;{\rm{(in}}\;{\rm{ML)}}\\ {G}_{T}({z}_{k})\;{\rm{or}}\;{G}_{D}({z}_{k})\;{\rm{evident}}\;{\rm{for}}\;{z}_{k} < -\,{h}_{T}\;{\rm{(in}}\;{\rm{thermocline)}}\;{\rm{or}}\;{z}_{k} < -\,{h}_{D}\;{\rm{(in}}\;{\rm{pycnocline)}}\end{array}\right.$$

Two types of methodology, single near-zero gradient and double gradients, are available to determine *h*_*T*_ and *h*_*D*_ from vertical *T* and ρ profiles. The single near-zero gradient method requires either the deviation of *T* (or *ρ*) from its value near the surface (i.e., reference level) to be smaller than a certain fixed value, such as 0.8 °C^9^, 0.2 °C^15,16^ to 0.1 °C^17^ for *T*, (0.03 kg/m^3^)^15^, (0.125 kg/m^3^)^18^, to (0.05 kg/m^3^)^19^ for *ρ*, or the near-zero vertical gradient to be smaller than a certain fixed value, such as (0.025 °C/m)^6,10^, (0.02 °C/m)^20^, to (0.015 °C/m)^21^ for *G*_*T*_(*z*_*k*_). To eliminate or reduce such uncertainty, the split-and-merge (SM)^22^, maximum curvature^23–25^, optimal linear fitting^26^, and maximum angle^27^ methods have been developed. The double gradient method is based on the transition of a *near-zero* gradient in the ITL (or ML) to an evident gradient in the thermocline (or pycnocline). The double gradient method with the exponential leap-forward gradient (ELG)^28,29^ was used in this study.

After (*h*_*T*_, *h*_*D*_) are determined from individual (*T*, *ρ*) profiles, the barrier (or compensated) layer depth, ocean heat content (OHC) for ITL (*H*_*ITL*_)^30^, freshwater content (FWC) for ML (*F*_*ML*_) were calculated. Note that *H*_*ITL*_ (or *F*_*ML*_) is the vertical integration of temperature (salinity) profile from the surface (z = 0) down to the base of the ITL (*z* = −*h*_*T*_) [or ML (*z* = −*h*_*D*_)] rather than to fixed depths such as (*H*_700_, *F*_700_) for the upper 700 m. Obviously, *H*_*ITL*_ and *F*_*ML*_ are new and different from traditionally defined OHC (or FWC) in the oceanographic community with fixed depth intervals from the ocean surface such as 0–150 m (in the Indian Ocean)^31^, 0–300 m^32^, 0–400 m^33^, 0–700 m^34^, 0–750 m^35^, 0–2000 m^36^, deep layer OHC such as below 2000 m^37^, and the full layer OHC^38^. Interested readers are referred to two excellent review papers^39,40^.

From vertical gradient data below ITL (or ML) *G*_*T*_(*z*_*k*_), *z*_*k*_ < −*h*_*T*_ [*G*_*D*_(*z*_*k*_), *z*_*k*_ < −*h*_*D*_], other thermohaline parameters can be identified from each *T*-profile (ρ-profile), such as maximum temperature (density) gradient, thermocline (pycnocline) depth, temperature (density) at thermocline (pycnocline) depth. The quality of the (*h*_*T*_, *h*_*D*_) data are estimated by the identification index^29^ (*i*-index, see Error Estimation Section). Altogether, this dataset, containing 17 thermohaline parameters with *i*-index, is located at the NCEI website (Data Citation 1) for public use.

Global climatological (annual mean and monthly mean) data of ocean thermohaline parameters can be established through two approaches: (1) analysing climatological (*T*, *S*) profiles such as earlier version of WOA18 to obtain climatological (*h*_*T*_, *h*_*D*_) data^5,9,13,41^, and (2) analysing observational profiles to get synoptic thermohaline parameters^29,30^ and then using optimal interpolation^42^, Kalmen filter^43^, or optimal spectral decomposition^44^ to produce gridded climatological thermohaline parameters. At present, we cannot estimate how big the difference is if using these two approaches. We can estimate only after the two approaches have been used. In this study, we take the first approach to derive climatological thermohaline parameter data from the NOAA/NCEI World Ocean Atlas 2018 (WOA18)^45^ annual and monthly mean temperature and salinity (*S*) profiles with regular 102 vertical levels (Table 1).

**Table 1:** Standard vertical depths of WOA18 data.

Standard Level	Standard Depth (m)	Standard Level	Standard Depth (m)	Standard Level	Standard Depth (m)	Standard Level	Standard Depth (m)
1	0	27	250	53	1300	79	3200
2	5	28	275	54	1350	80	3300
3	10	29	300	55	1400	81	3400
4	15	30	325	56	1450	82	3500
5	20	31	350	57	1500	83	3600
6	25	32	375	58	1550	84	3700
7	30	33	400	59	1600	85	3800
8	35	34	425	60	1650	86	3900
9	40	35	450	61	1700	87	4000
10	45	36	475	62	1750	88	4100
11	50	37	500	63	1800	89	4200
12	55	38	550	64	1850	90	4300
13	60	39	600	65	1900	91	4400
14	65	40	650	66	1950	92	4500
15	70	41	700	67	2000	93	4600
16	75	42	750	68	2100	94	4700
17	80	43	800	69	2200	95	4800
18	85	44	850	70	2300	96	4900
19	90	45	900	71	2400	97	5000
20	95	46	950	72	2500	98	5100
21	100	47	1000	73	2600	99	5200
22	125	48	1050	74	2700	100	5300
23	150	49	1100	75	2800	101	5400
24	175	50	1150	76	2900	102	5500
25	200	51	1200	77	3000		
26	225	52	1250	78	3100		

Furthermore, on the base of the single gradient method (i.e., near-zero gradient in the mixed layer), serval global climatological datasets of ocean (*h*_*T*_, *h*_*D*_) were produced from the earlier version of WOA18 such as the NOAA/NCEI Mixed Layer Depth Data^5^, the Naval Research Laboratory Mixed Layer Depth (NMLD) Climatologies^41^, and comprehensive Mixed Layer Data for the Indian Ocean^13^. All these datasets don’t include any parameters below the ITL/ML. On the base of the double-gradient method, this dataset^45^ contains more thermohaline parameters from WOA18 such as thermocline gradient (*G*_*T*_), pycnocline gradient (*G*_*D*_), ITL heat content (*H*_*ITL*_), mixed layer fresh-water content (*F*_*ML*_), maximum thermocline gradient (*G*_*Tmax*_), thermocline depth (*h*_*th*_), temperature at thermocline depth (*T*_*th*_), maximum density gradient *G*_*Dmax*_), pycnocline depth (*h*_*pyc*_), density at pycnocline depth (*ρ*_*pyc*_), and (*h*_*T*_ – *h*_*D*_) (barrier layer if positive or compensated layer if negative). Among them, ITL heat content (*H*_*ITL*_), and mixed layer fresh-water content (*F*_*ML*_) are different from the commonly used fixed-depth heat and freshwater contents.

## Methods

These methods can be found in our related work^28,29^.

### Main part of pycnocline (or Thermocline)

**Fig. 2:**
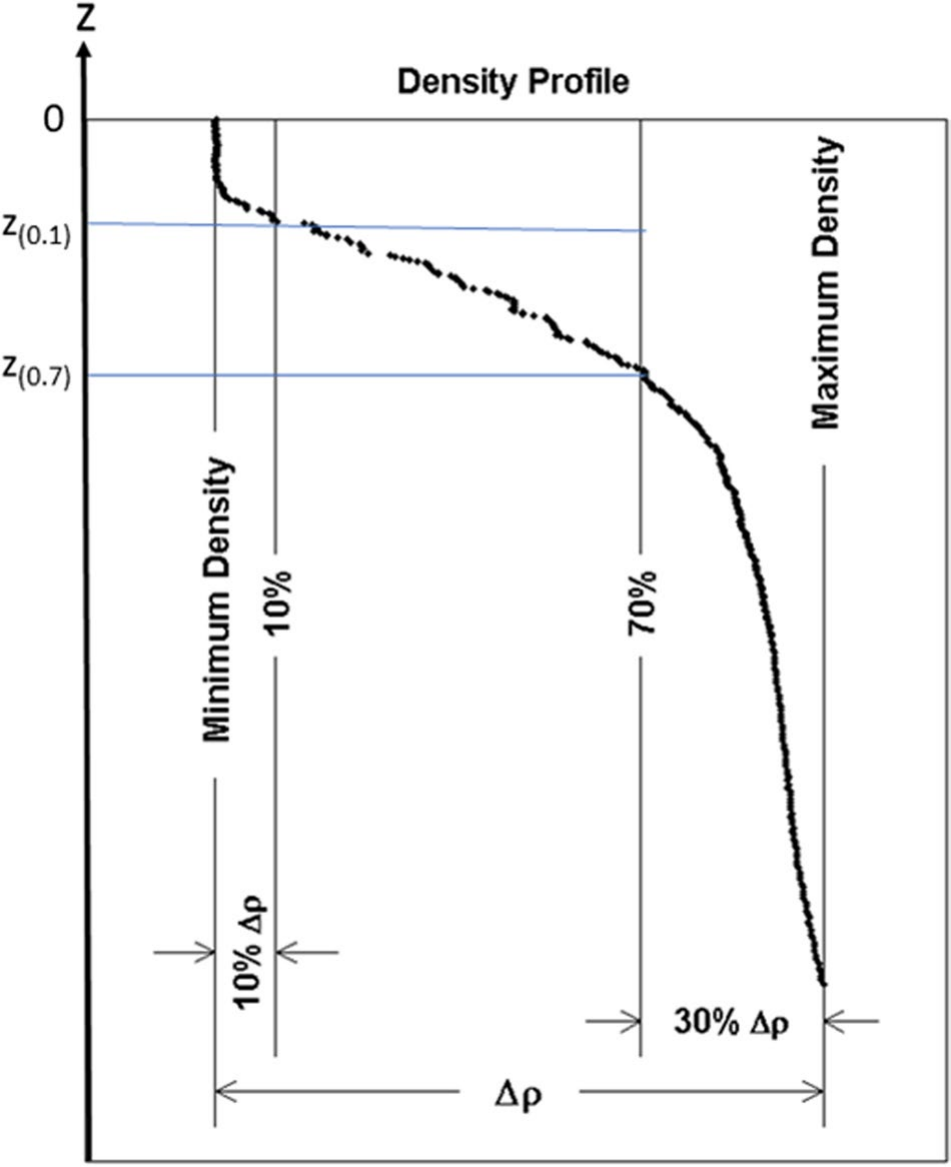
Illustration for determination of *z*_(0.1)_ and *z*_(0.7)_ for *ρ*-profile.

The ρ-profile [*ρ*(*z*_k_)] is taken for illustration. Let the depths corresponding to *ρ*_min_ and *ρ*_*max*_ be *z*_*1*_ and *z*_*K*_. The vertical density difference, Δ*ρ* = *ρ*_*max*_ − *ρ*_min_, represents the total variability. Theoretically, the variability is 0 in ML and large in pycnocline beneath the ML. It is reasonable to identify the main part of the pycnocline between the two depths: z_(0.1)_ and *z*_(0.7)_, with the difference to *ρ*_min_ as 0.1Δ*ρ* and 0.7Δ*ρ* (Fig. 2), respectively. Here, *ρ*(*z*_(0.1)_) = *ρ*_min_ + 0.1Δ*ρ*, and *ρ*(*z*_(0.7)_) = *ρ*_min_ + 0.7Δ*ρ*.

### Pycnocline and thermocline gradients

The data between z_(0.1)_ and *z*_(0.7)_ is rearranged into [ρ_*i*_, *i* = 0, 1, 2, …, *I*] with [*ρ*_0_ = *ρ*(*z*_(0.1)_), *ρ*_*I*_ = *ρ*(*z*_(0.7)_)]. Vertical gradients are calculated between *ρ*_*i*_ (*i* = 1, 2, …, *I*) and *ρ*_0_,

2 $${G}_{Di}=-\frac{\rho ({z}_{(0.1)})-{\rho }_{i}}{{z}_{(0.1)}-{z}_{i}},\quad {\rho }_{i}=\rho {{\rm{(z}}}_{i}{\rm{),}}\;i=1,2,\ldots ,I$$

**Fig. 3:**
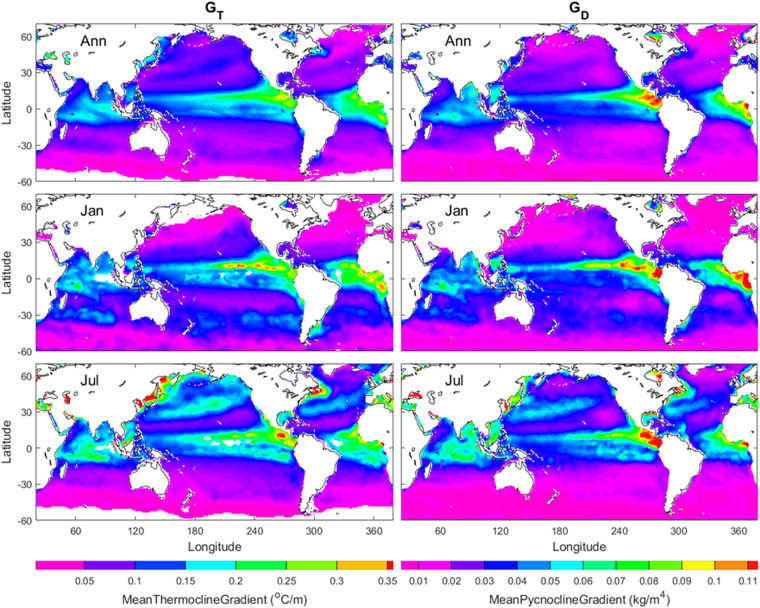
Annual, January, and July mean (*G*_*T*_, *G*_*D*_) maps with the left panels for *G*_*T*_ and the right panels for *G*_*D*_. The areas in open oceans with the white colour indicate low quality of the identification. The land and the areas of low quality of the identification are represented by white with the land enclosed by black curves (i.e., coasts).

Their median

3 $${G}_{D}={\rm{Median}}\left\{{G}_{D1},{G}_{D2},\ldots ,{G}_{DI}\right\}$$

is used to represent the *characteristic gradient* for the pycnocline, and it is simply called the pycnocline gradient (*G*_*D*_). Similarly, the same procedure id used to obtain the thermocline gradient (*G*_*T*_). The annual mean (*G*_*T*_, *G*_*D*_) maps show gradients are stronger in tropical regions (20°S–20°N) than middle and high latitudes. In the low latitudes, the gradients are stronger in the eastern than western Pacific and Atlantic. The January and July mean (*G*_*T*_, *G*_*D*_) maps show stronger seasonal variability in the northern hemisphere than in the southern hemisphere (Fig. 3).

### ELG for determining MLD (ILD)

**Fig. 4:**
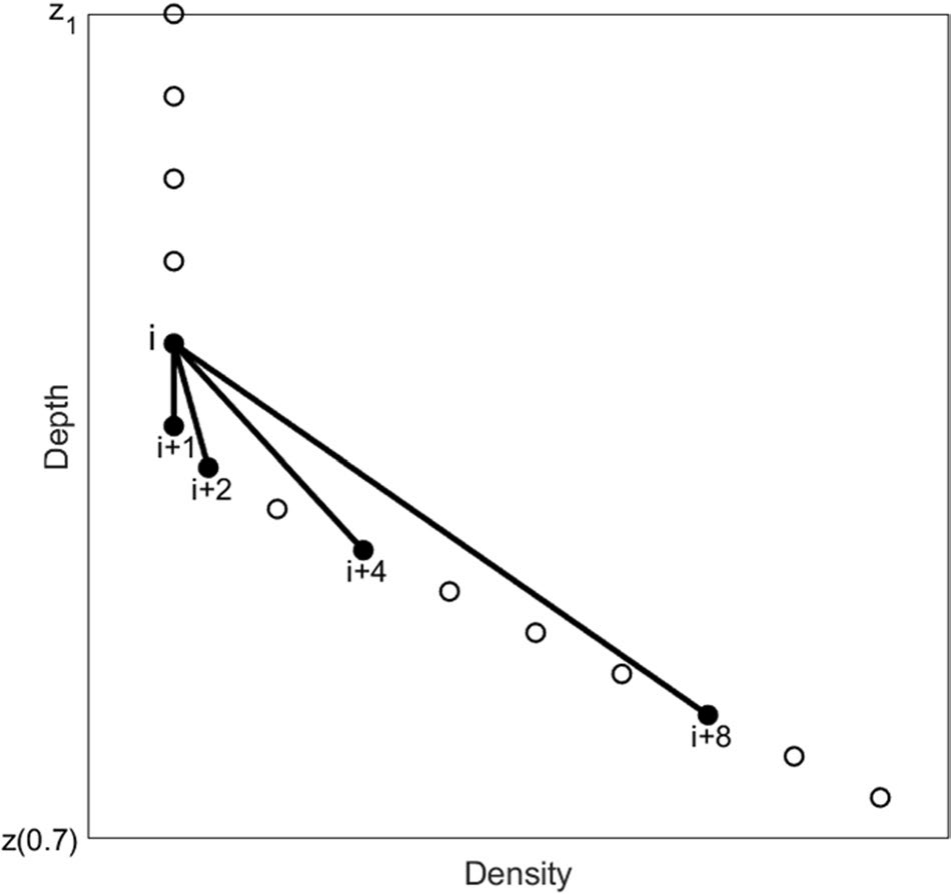
Illustration of the exponentially leap-forward gradient (ELG) method.

Go back to the original profile shown in Fig. 2. Let the number of the data points between *z*_1_ and *z*_(0.7)_ be *N*_*g*_, and let *N* = [log_2_(*N*_*g*_)] with the bracket indicating the integer part of the real number inside. *N* is much smaller than *N*_*g*_. Starting from *z*_1_, the (*N* + 1) exponential leap-forward gradients (ELGs) are calculated at depth *z*_*k*_ [between *z*_1_ and z_(0.7)_] (Fig. 4)

4 $${D}_{n}\rho ({z}_{k})=\frac{\rho \left({z}_{k}\right)-\rho \left({z}_{k+{2}^{n}}\right)}{{z}_{k}-{z}_{k+{2}^{n}}},\;n=0,1,2,\ldots ,N$$

where *D*_*n*_ is the difference operator: $${D}_{1}\rho ({z}_{k})=[\rho ({z}_{k})-\rho ({z}_{k+2})]/({z}_{k}-{z}_{k+2}),$$ $${D}_{2}\rho ({z}_{k})\,=\,$$$$[\rho ({z}_{k})-\rho ({z}_{k+4})]/({z}_{k}-{z}_{k+4}),$$ etc. . The averaged value among (*N* + 1) gradients [*D*_0_*ρ*(*z*_*k*_), *D*_1_*ρ*(*z*_*k*_), …, *D*_*N*_*ρ*(*z*_*k*_)] is computed by

5 $${\widetilde{G}}_{D}\left({z}_{k}\right)=-\frac{\mathop{\sum }\limits_{n=0}^{N}{D}_{n}T({z}_{k})}{N+1}$$

which represents the gradient effectively at the depth *z*_*k*_ with capability to filter out noises in the gradient calculation^28,29^.

**Fig. 5:**
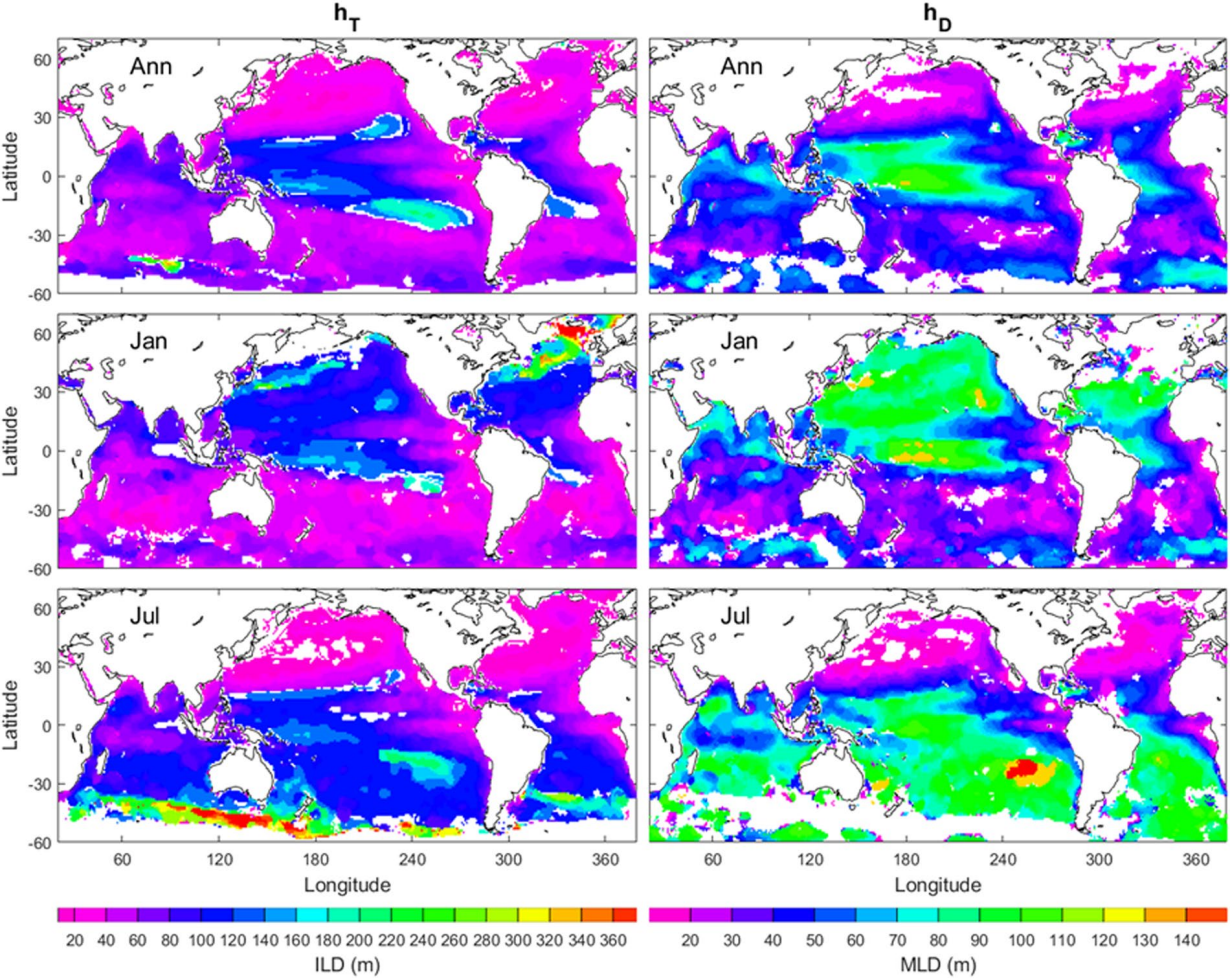
Annual, January, and July mean (*h*_*T*_, *h*_*D*_) maps with the left panels for *h*_*T*_ and the right panels for *h*_*D*_. The areas in open oceans with the white colour indicate low quality of the identification. The land and the areas of low quality of the identification are represented by white with the land enclosed by black curves (i.e., coasts). The red bull-eye in the southeast Pacific in the right-lower panel indicates the area of *h*_*D*_ = 150 m.

Since ${\widetilde{G}}_{D}\left({z}_{k}\right)\approx 0$ if *z*_*k*_ in the mixed layer; ${\widetilde{G}}_{D}({z}_{k})={G}_{pyc}$ if *z*_*k*_ in the pycnocline, it is reasonable to use (an order of smaller gradient in mixed layer than in pycnocline),

6 $${\widetilde{G}}_{D}\left({z}_{k}\right)/{G}_{pyc} < 0.1,\quad {z}_{k}\;{\rm{in}}\;{\rm{mixed}}\;{\rm{layer}}$$

to identify *h*_*D*_ (similarly *h*_*T*_). The annual mean (*h*_*T*_, *h*_*D*_) maps (upper panels in Fig. 5) show deeper ILD and MLD in the western than eastern Pacific and Atlantic in low latitudes (30°S–30°N). The January and July mean (*h*_*T*_, *h*_*D*_) maps (middle and lower panels in Fig. 5) show stronger seasonal variability with deeper (*h*_*T*_, *h*_*D*_) in January (July) in the northern (southern) hemisphere, deeper (*h*_*T*_, *h*_*D*_) in the Gulf Stream and Kuroshio areas in January, deeper (*h*_*T*_, *h*_*D*_) in east of Southern Pacific in July, and deeper *h*_*T*_ in the Circumpolar current west of the Drake Passage in July.

### Barrier (Compensated) layer

**Fig. 6:**
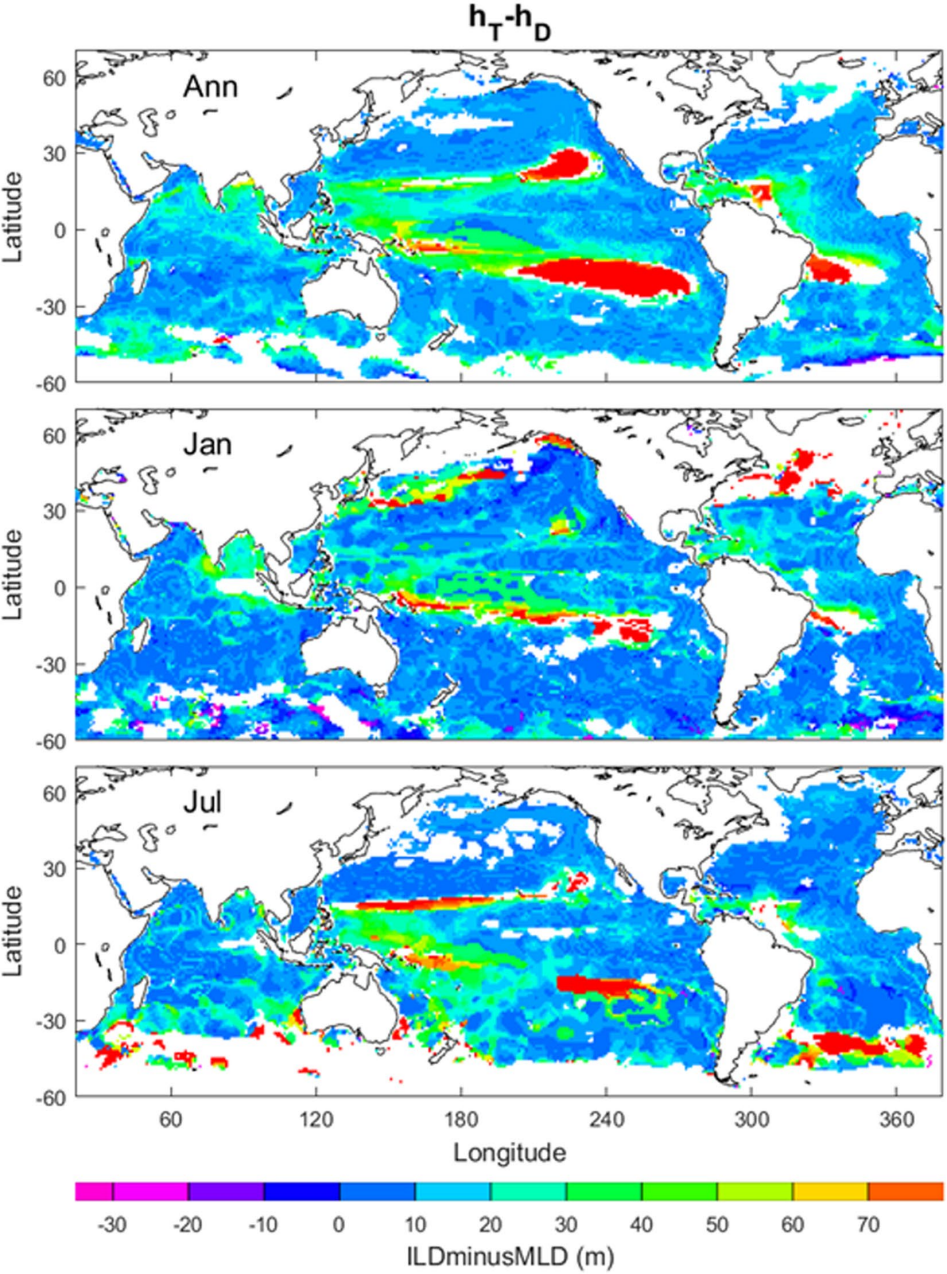
Annual, January, July mean (*h*_*T*_ − *h*_*D*_) maps. The areas in open oceans with the white colour indicate low quality of the identification. The land and the areas of low quality of the identification are represented by white with the land enclosed by black curves (i.e., coasts).

Barrier layer occurs if *h*_*T*_ > *h*_*D*_, with the barrier layer depth (BLD) of (*h*_*T*_ − *h*_*D*_). Compensated layer occurs if *h*_*T*_ < *h*_*D*_, with the compensated layer depth (CLD) of (*h*_*D*_ − *h*_*T*_)^15^. We generate the difference (*h*_*T*_ − *h*_*D*_) data to identify the occurrence of barrier or compensated layer, with annual, January, July mean (*h*_*T*_ − *h*_*D*_) maps. The occurrence of barrier layer is much often than the occurrence of compensated layer with the ratio of 23562/170 (~139) from the annual data. Evident barrier layer occurs in the extra-tropical (20°–30°N, 10°–28°S) eastern Pacific Ocean, tropical (0°–8°S) western Pacific west of New Guinea, and low latitudinal Brazilian coast in the annual mean; Kuroshio and Gulf Stream extension regions in January; and south (30°–40°S) Southern Atlantic Ocean in July (Fig. 6).

### ITL temperature and ML density

The ITL temperature is the vertically averaged temperature from the sea surface (*z* = 0) down to ILD (*z* = − *h*_*T*_),

7 $${T}_{ITL}=\frac{1}{{h}_{T}}\underset{-{h}_{T}}{\overset{0}{\int }}Tdz$$

The ML density is the vertically averaged *ρ* from the sea surface (*z* = 0) down to MLD (*z* = −*h*_*D*_)

8 $${\rho }_{ML}=\frac{1}{{h}_{D}}\underset{-{h}_{D}}{\overset{0}{\int }}\rho dz$$

**Fig. 7:**
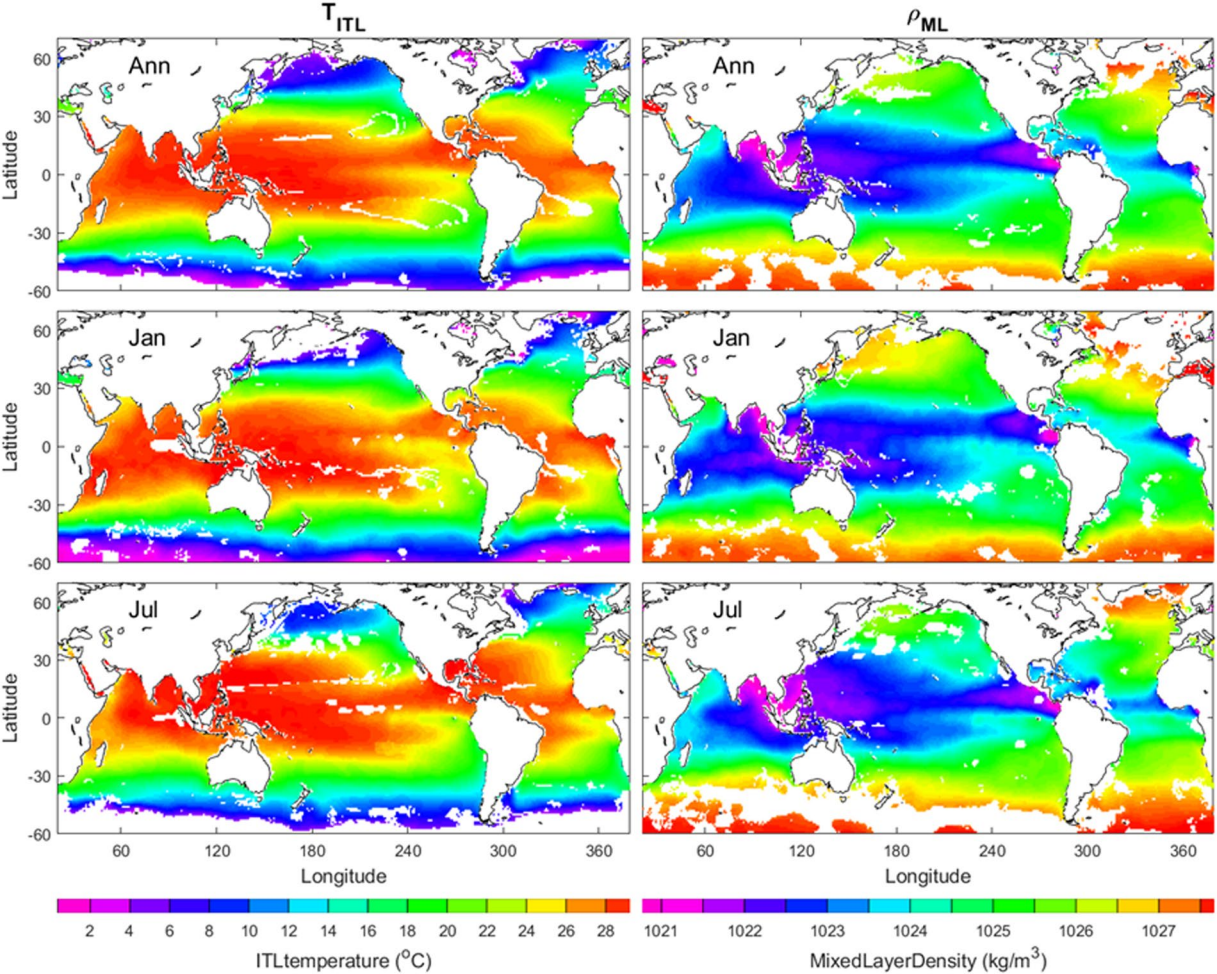
Annual, January, July mean (*T*_*ITL*_, *ρ*_*ML*_) with the left panels for *T*_*ITL*_ and the right panels for *ρ*_*ML*_. The land and the areas of low quality of the identification are represented by white with the land enclosed by black curves (i.e., coasts).

Figure 7 shows the annual, January, July mean (*T*_*ITL*_, *ρ*_*ML*_). Note that (*T*_*ITL*_, *ρ*_*ML*_) are not the same as the sea surface temperature and density. The variables (*T*_*ITL*_, *ρ*_*ML*_) follow the ocean mixed layer dynamics. For example, *T*_*ITL*_ satisfies following equation due to the ITL layer heat balance^46^

9 $${h}_{T}\frac{\partial {T}_{ITL}}{\partial t}+{h}_{T}{\bf{V}}\,\bullet \,\nabla {T}_{ITL}+\Lambda {w}_{e}\Delta T+\nabla \,\bullet \,\underset{-{h}_{T}}{\overset{0}{\int }}\widehat{{\bf{V}}}\widehat{T}dz=\frac{{Q}_{0}-{Q}_{-{h}_{T}}}{\rho {c}_{p}},\quad \Delta T\equiv {T}_{ITL}-{T}_{th}$$

where **V** is the vertically averaged horizontal velocity from the surface to −*h*_*T*_; $\widehat{{\bf{V}}}$ and $\widehat{T}$ are deviation from the vertical average; *Q*_0_ is the net surface heat flux adjusted for the penetration of light below the ITL; ${{\rm{Q}}}_{-{{\rm{h}}}_{{\rm{T}}}}$ is the vertical turbulent diffusion at the base of ITL; *w*_*e*_ is the entrainment velocity; Λ is the Heaviside unit function taking 0 if *w*_*e*_ < 0, and 1 otherwise due to the second law of the thermodynamics; Δ*T* is the temperature difference between ITL and thermocline; *T*_*th*_ is the thermocline temperature.

### ITL heat content and ML freshwater content

**Fig. 8:**
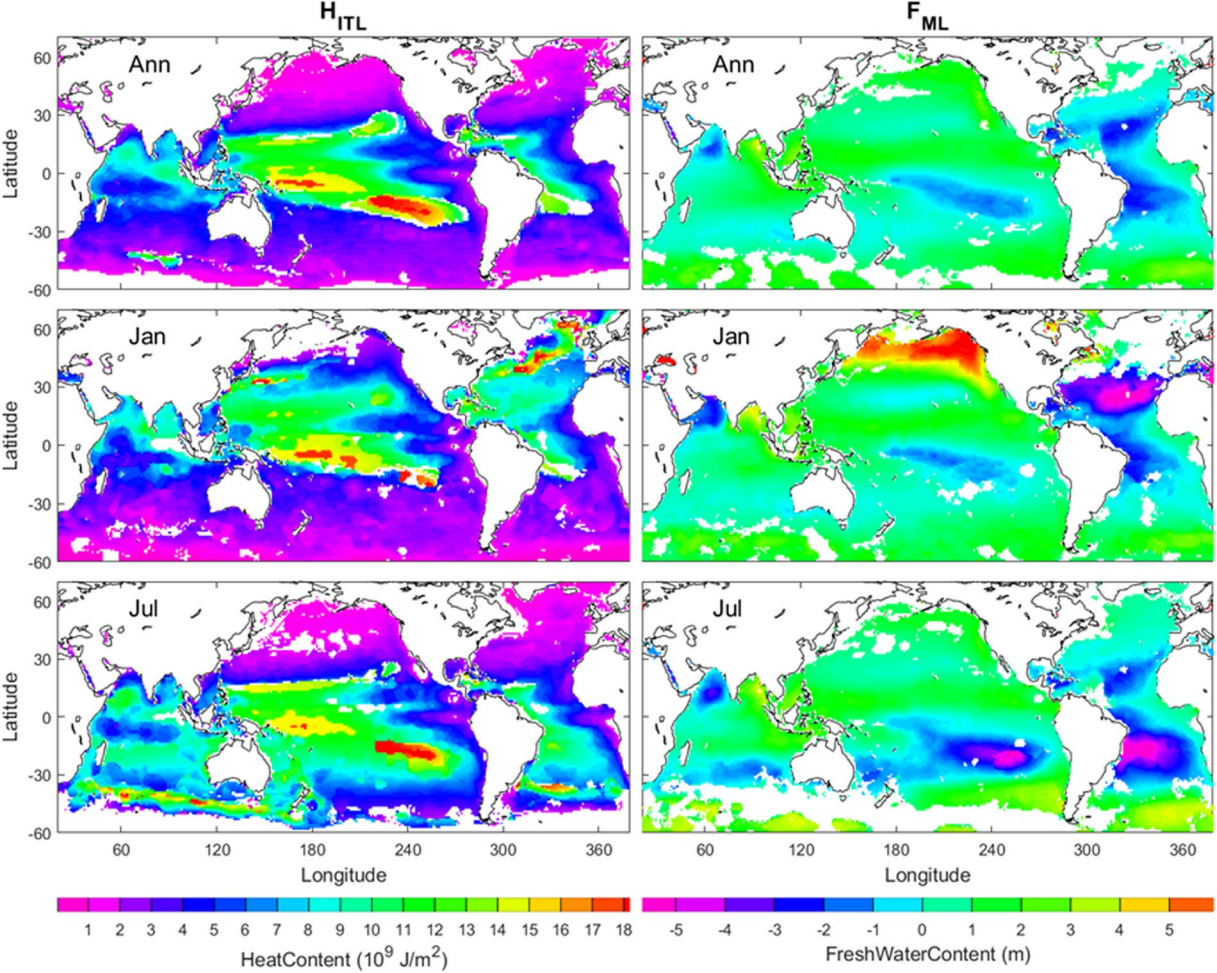
Annual, January, July mean (*H*_*ITL*_, *F*_*ML*_) maps with the left panels for *H*_*ITL*_ and the right panels for *F*_*ML*_. The land and the areas of low quality of the identification are represented by white with the land enclosed by black curves (i.e., coasts).

The ITL heat content is the integrated heat stored in the layer from the sea surface (*z* = 0) down to ILD (*z* = − *h*_*T*_),

10 $${H}_{ITL}={c}_{p}\underset{-{h}_{T}}{\overset{0}{\int }}\rho (z)T(z)dz$$

where *c*_*p*_ = 3,985 J kg^−1^ °C^−1^, is the specific heat for sea water. The annual, January, and July mean *H*_*ITL*_ show that *H*_*ITL*_ is higher in low latitudes than in middle and high latitudes and is higher in the western than eastern Pacific and Atlantic Oceans within the low latitudes (left panels in Fig. 8). The ITL heat content *H*_*ITL*_ represents the warming/cooling of the ITL only. However, the fixed-depth OHC represents warming/cooling of ITL, combined ITL-part of thermocline, or combined ITL-thermocline-part of deep layer depending on the selection of depth with various warming trends. For example, the warming trend is estimated as 0.64 ± 0.11 W m^−2^ for OHC (0–300 m) in 1993–2008^32^, and 0.20 W m^−2^ for OHC (0–700 m) in 1955–2012^36^. The global ocean ITL warming rate from 1970 to 2017 is identified as (0.14 W m^−2^)^47^.

The ML freshwater content (*F*_*ML*_) is the integrated heat stored in the layer from the sea surface (*z* = 0) down to MLD (*z* = − *h*_*D*_)^48^,

11 $${F}_{ML}=\underset{-{h}_{D}}{\overset{0}{\int }}\left[1-\frac{S(z)}{{S}_{ref}}\right]dz,\;\;{S}_{ref}={\rm{35}}\;{\rm{psu}}$$

The annual mean *F*_*ML*_ is mostly positive in the North Pacific Ocean, the Southern Ocean, the Indian Ocean except the Arabian Sea, and mostly negative in the Atlantic Oceans. Four evident negative *F*_*ML*_ areas are in the Arabian Sea, central and eastern South Pacific Ocean between equator to 25°S, the North Atlantic Ocean from the Caribbean Sea to the west coast of Spain, and the South Atlantic Ocean between the equator to 30°S (upper right panel in Fig. 8).

Seasonal variability is evident with stronger positive *F*_*ML*_ in the North Pacific Ocean, weaker negative *F*_*ML*_ in the South Pacific Ocean, stronger negative *F*_*ML*_ in the North Atlantic Ocean, weaker negative *F*_*ML*_ in the South Atlantic Ocean in January than in July (central and lower right panels in Fig. 8). Strongest positive *F*_*ML*_ (>5 m) occurs in the northern (35° N–60° N) North Pacific Ocean in January. Strongest negative *F*_*ML*_ (<−5 m) occurs in the central to eastern subtropical North Atlantic Ocean in January, and in the eastern subtropical South Pacific Ocean and the subtropical South Atlantic Ocean in July. Note that the ML freshwater content (*F*_*ML*_) dataset is also new since it is different from the fixed-depth freshwater content.

### Thermocline and pycnocline

Maximum gradients can be obtained from vertical gradients of (*T*, *ρ*) between *z*_(0.1)_ and *z*_(0.7)_ calculated by Eq. (2),

12 $${G}_{T\max }={\rm{Max}}\left({G}_{T1},{G}_{T2},\ldots ,{G}_{TI}\right),\;{G}_{D\max }={\rm{Max}}\left({G}_{D1},{G}_{D2},\ldots ,{G}_{DI}\right)$$

which are used to represent the depths of thermocline (*h*_*th*_) and pycnocline (*h*_*pyc*_)

13 $${G}_{Ti}\left({z}_{i}=-\,{h}_{th}\right)={G}_{T\max },\;{G}_{Di}\left({z}_{i}=-\,{h}_{pyc}\right)={G}_{D\max }$$

The temperature at the thermocline depth is defined as the thermocline temperature

14 $${T}_{th}=T\left(z=-\,{h}_{th}\right)$$

The density at the pycnocline depth is defined as the pycnocline density

15 $${\rho }_{pyc}=\rho \left(z=-\,{h}_{pyc}\right)$$

**Fig. 9:**
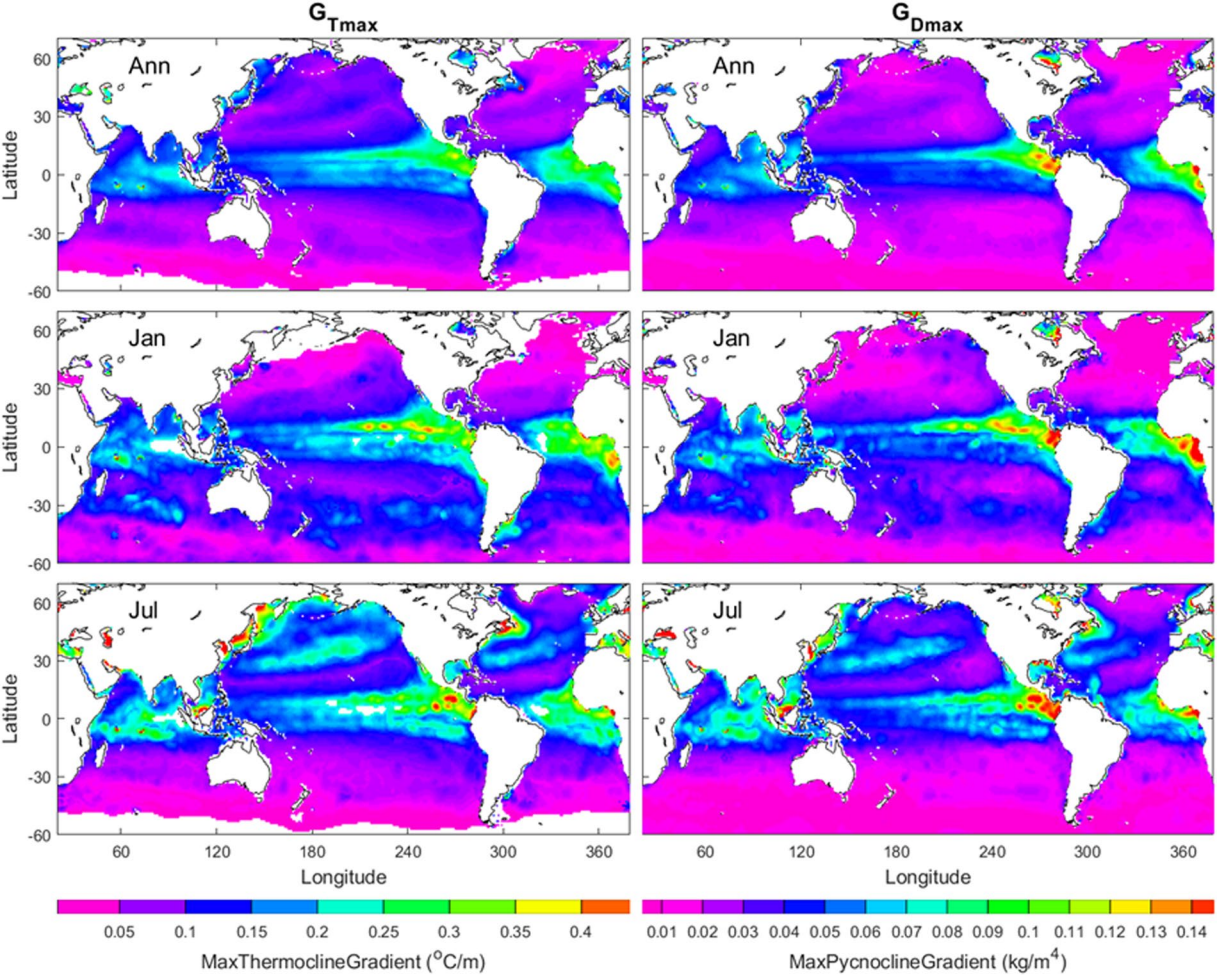
Maps of annual, January, July mean (*G*_*Tmax*_, *G*_*Dmax*_).

Annual mean *G*_*Tmax*_ and *G*_*Dmax*_ have evident latitudinal variability with large values in the tropical regions and small values outside the tropical region (upper two panels in Fig. 9). In the tropical Pacific, Atlantic, and Indian Oceans, the large values are in the central and eastern parts. Seasonal variability of (*G*_*Tmax*_, *G*_*Dmax*_) is strong in the Northern Hemisphere and weak in the Southern Hemisphere. In the Northern Hemisphere, the difference is small between annual and January mean (*G*_*Tmax*_, *G*_*Dmax*_) but large between annual and July mean (*G*_*Tmax*_, *G*_*Dmax*_). In July, very strong (*G*_*Tmax*_, *G*_*Dmax*_) occur in the northeastern Asian and American coastal regions, and strong (*G*_*Tmax*_, *G*_*Dmax*_) appear in the Kuroshio extension and Gulf Stream regions (middle and lower panels in Fig. 9).

**Fig. 10:**
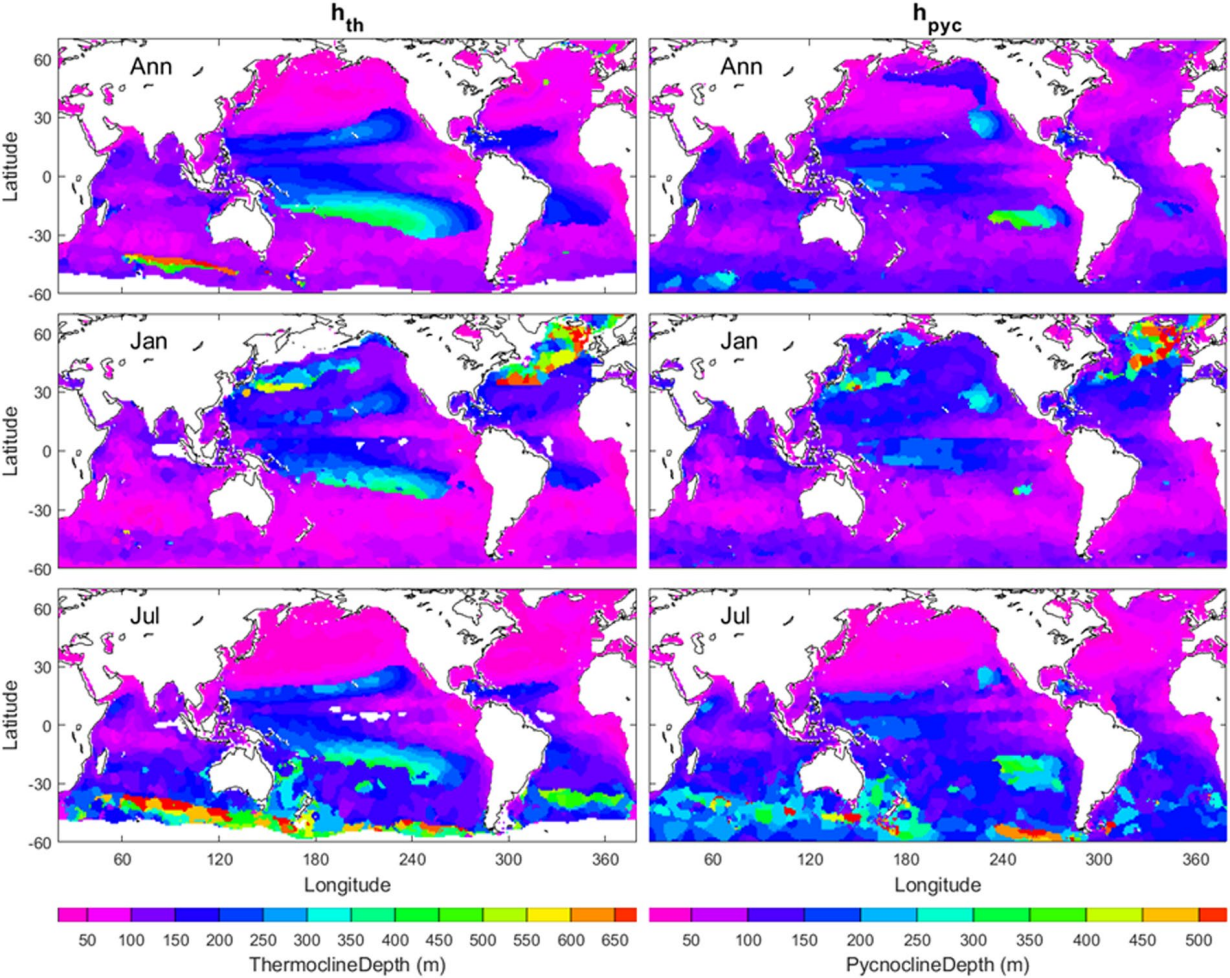
Maps of annual, January, July mean (*h*_*th*_, *h*_*pyc*_) with the left panels for *h*_*th*_ and the right panels for *h*_*pyc*_. The land and the areas of low quality of the identification are represented by white with the land enclosed by black curves (i.e., coasts). The “slice-like” feature may be caused by the vertical resolution of 25 m from 100 m to 500 m depth in the WOA18.

Annual mean *h*_*th*_ and *h*_*pyc*_ have evident spatial variability with large values in low latitudes (30°S–30°N) with shallowing of (*h*_*th*_, *h*_*pyc*_) in the eastern Pacific and Atlantic Oceans (upper two panels in Fig. 10). Seasonal variability of (*h*_*th*_, *h*_*pyc*_) is strong in middle and high latitudes with deep (*h*_*th*_, *h*_*pyc*_) in (30°N–60°N) in January and deep (*h*_*th*_, *h*_*pyc*_) in (30°S–60°S) in July (middle and lower panels in Fig. 10).

**Fig. 11:**
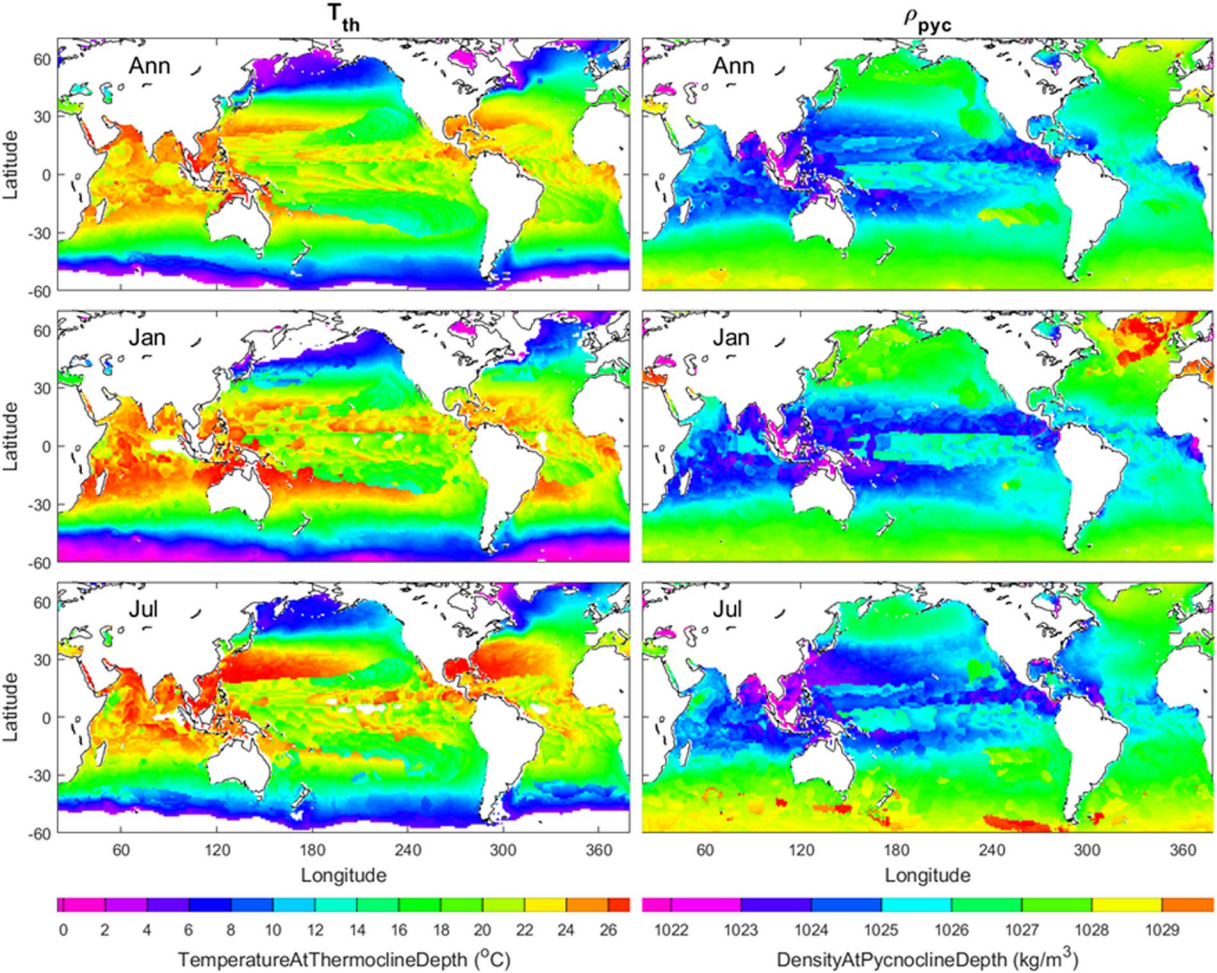
Maps of annual, January, July mean (*T*_*th*_, *ρ*_*pyc*_) with the left panels for *T*_*th*_ and the right panels for *ρ*_*pyc*_. The land and the areas of low quality of the identification are represented by white with the land enclosed by black curves (i.e., coasts). The “slice-like” feature may be caused by the vertical resolution of 25 m from 100 m to 500 m depth in the WOA18.

Annual mean *T*_*th*_ and *ρ*_*pyc*_ have evident spatial variability with warm *T*_*th*_ and light *ρ*_*pyc*_ in low latitudes (30°S–30°N) and cold *T*_*th*_ and dense *ρ*_*pyc*_ in middle and high latitudes (30°S–60°S, 30°N–60°N) (upper two panels in Fig. 11). Seasonal variability of (*T*_*th*_, *ρ*_*pyc*_) is generally weak except in subtropical oceans where warmer *T*_*th*_ (light *ρ*_*pyc*_) in the Sothern Hemisphere in January and in the Northern Hemisphere in July. Furthermore, cold *T*_*th*_ and dense *ρ*_*pyc*_ appears in North Atlantic Ocean near Greenland in January (middle and lower panels in Fig. 11).

### Global statistics of the thermohaline parameters

The identified thermohaline parameters are on the grid points. We use the standard Matlab codes to calculate with area-weighted mean, standard deviation, skewness, and kurtosis for each parameter. Table 2 shows the statistical characteristics of thermohaline parameters derived from the annual mean (*T*, *ρ*) profiles. These values can be treated as the overall climatological values of global thermohaline parameters, such as 51.4 m for isothermal layer depth, 38.7 m for mixed layer depth, 14.3 m for barrier layer depth, 99.4 m the thermocline depth, 73.9 m for the pycnocline depth, 0.0638 °C/m for thermocline gradient, and 0.0212 kg/m^4^ for pycnocline gradient.

**Table 2:** Statistical characteristics of thermohaline parameters derived from the annual mean (*T*, *ρ*) profiles.

Thermohaline Parameter	Mean	Standard Deviation	Skewness	Kurtosis
Isothermal Layer Depth (m)	51.4	34.2	2.16	11.3
Mixed Layer Depth (m)	38.7	19.1	0.98	3.95
Mean Thermocline Gradient (°C/m)	0.0638	0.0501	1.49	5.17
Mean Pycnocline Gradient (kg/m^4^)	0.0212	0.0179	2.36	12.3
Barrier Layer Depth (m) (Total #: 23562)	14.3	22.4	3.70	18.6
Compensated Layer Depth (m) (Total #: 170)	11.3	12.5	3.33	15.2
Isothermal Layer Heat Content (10^9 ^J/m^2^)	4.30	3.80	1.41	4.39
Isothermal Layer Temperature (°C)	19.0	8.02	−0.595	2.02
Maximum Thermocline Gradient (°C/m)	0.0742	0.0580	1.61	6.98
Thermocline Depth (m)	99.4	85.2	2.97	15.1
Temperature at Thermocline Depth (°C)	15.6	6.62	−0.624	2.27
Mixed Layer Freshwater Content (m)	0.0932	0.987	−0.310	2.90
Mixed Layer Density (kg/m^3^)	1024.60	1.74	−0.636	8.10
Maximum Pycnocline Gradient (kg/m^4^)	0.0246	0.023	3.4	27.3
Pycnocline Depth (m)	73.9	39.4	2.20	13.1
Density at Pycnocline Depth (kg/m^3^)	1025.97	1.56	−2.17	25.7

## Data Records

This global ocean climatology of thermohaline parameter dataset is publicly available at the NOAA/NCEI data repository as a NetCDF file, which includes data citation, dataset identifiers, metadata, and ordering instructions. The dataset is located at the NOAA/NCEI website (10.25921/j3v2-jy50).

## Technical Validation

From the identified parameters (*h*_*T*_, *G*_*T*_) and (*h*_*D*_, *G*_*D*_), fitted temperature and density profiles [$\widehat{T}({z}_{k}),\widehat{\rho }({z}_{k})$] can be constructed using zero gradient in the ITL and ML, and linear gradient (*G*_*T*_, *G*_*D*_) in the thermocline and pycnocline,

16a $$\widehat{T}({z}_{k})=\left\{\begin{array}{l}{T}_{ITL}\quad \quad {\rm{for}}\;{z}_{k}\ge -\,{h}_{T}\;{\rm{(in}}\;{\rm{ITL)}}\\ {T}_{ITL}+({z}_{k}+{h}_{T}){G}_{T}\quad {\rm{for}}\;{z}_{k} < -\,{h}_{T}\;{\rm{(in}}\;{\rm{thermocline)}}\end{array}\right.$$

16b $$\widehat{\rho }({z}_{k})=\left\{\begin{array}{l}{\rho }_{ML}\quad \quad {\rm{for}}\;{z}_{k}\ge -\,{h}_{D}\;{\rm{(in}}\;{\rm{ML)}}\\ {\rho }_{ML}-({z}_{k}+{h}_{D}){G}_{D}\quad {\rm{for}}\;{z}_{k} < -\,{h}_{T}\;{\rm{(in}}\;{\rm{pycnocline)}}\end{array}\right.$$

The methodology is evaluated through the comparison between the fitted profiles [$\widehat{T}({z}_{k}),\widehat{\rho }({z}_{k})$] and the WOA18 profiles [$T({z}_{k}),\rho ({z}_{k})$].

**Fig. 12:**
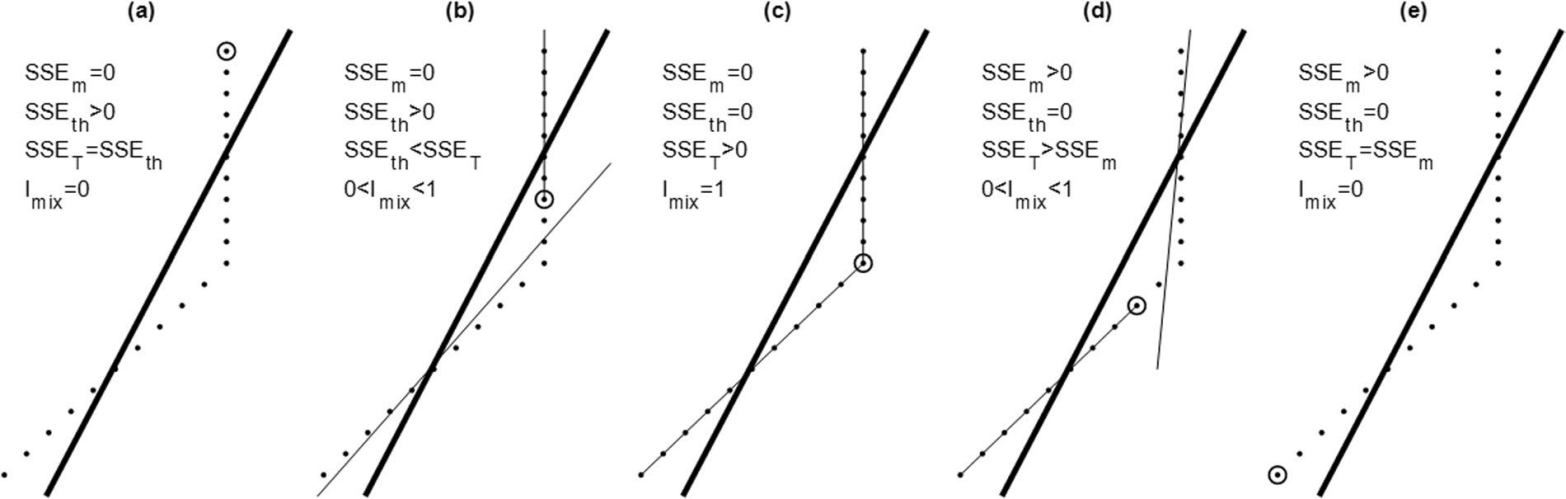
The i-index to represent the quality of the ITL depth determination. (**a**) ITL not existence or ITL in existence, but not identified (*h*_*T*_ = 0). (**b**) Identified ITL depth shallower than the real ITL depth. (**c**) Perfectly identified ITL depth. (**d**) Identified ITL depth deeper than the real ITL depth. (**e**) Identified ITL depth at *z*_(0.7)_, i.e., thermocline not existence. Here, the ITL depth is marked by a circle. The thick line in each figure is a single linear function fitted to the temperature profile data.

We take *T*-profile for illustration. The fitted $\widehat{T}({z}_{k})$ profile is represented by two lines with the first one (near zero gradient in the ITL) from the top to the ITL base (circles in Fig. 12) and the second one (non-zero gradient in the thermocline) from the ITL base to the bottom of the profile. The quality in determination of (*h*_*T*_, *G*_*T*_) is identified by the sum of the square error (SSE) between the fitted $\widehat{T}({z}_{k})$ and WOA18 *T*(*z*_*k*_) at the WOA18 vertical levels (see Table 1),

17 $$SSE=\sum _{k}{\left[\widehat{T}({z}_{k})-T({z}_{k})\right]}^{2}$$

Such double-gradient fitting has errors in the ITL represented by the sum of the square error in ITL (SSE_ITL_) and in the thermocline represented by the sum of the square error in thermocline (SSE_TH_). The whole *T*-profile data fitted to a single linear function (thick lines in Fig. 12) represents the maximum error since it disregards the existence of ITL and thermocline. Such a maximum error is represented by the total sum of the square error (SSE_T_). The identification index (called *i*-index) is defined by^29^

18 $${I}_{ITL}=\sqrt{1-\left(SS{E}_{ITL}+SS{E}_{TH}\right)/SS{E}_{T}}.$$

If an ITL exists but is not identified (*h*_*T*_ = 0) (Fig. 12a), SSE_ITL_ = 0, SSE_TH_ = SSE_T_; which gives *I*_*ITL*_ = 0. If the identified *h*_*T*_ is shorter than the real one (Fig. 12b), SSE_ITL_ = 0, SSE_TH_ > 0, SSE_TH_ < SSE_T_; which leads to 0 < *I*_*ITL*_ < 1. If the identified *h*_*T*_ is the same as the real one (Fig. 12c), SSE_ITL_ = 0, SSE_TH_ = 0, SSE_T_ > 0; which makes *I*_*ITL*_ = 1. If the identified *h*_*T*_ is deeper than the real one (Fig. 12d), SSE_ITL_ > 0, SSE_TH_ = 0, SSE_TH_ < SSE_T_; which leads to 0 < *I*_*ITL*_ < 1. If the identified *h*_*T*_ reaches the bottom of the thermocline (Fig. 12e), SSE_TH_ = 0, SSE_ITL_ = SSE_T_; which gives *I*_*ITL*_ = 0.

Thus, the value of *I*_*ITL*_ represents the quality of the determination of *h*_*T*_ from *T*-profile: (a) *I*_*ITL*_ = 1 for no error (Fig. 12c), (b) *I*_*ITL*_ = 0 for 100% error with no ITL identified but actual existence of ITL (Fig. 12a) or identified *h*_*T*_ at the bottom of the thermocline (Fig. 12e,c) 0 < *I*_*ITL*_ < 1 for identified *h*_*T*_ shallower than the actual *h*_*T*_ (Fig. 12b) and for identified *h*_*T*_ deeper than the actual *h*_*T*_ (Fig. 12d).

**Fig. 13:**
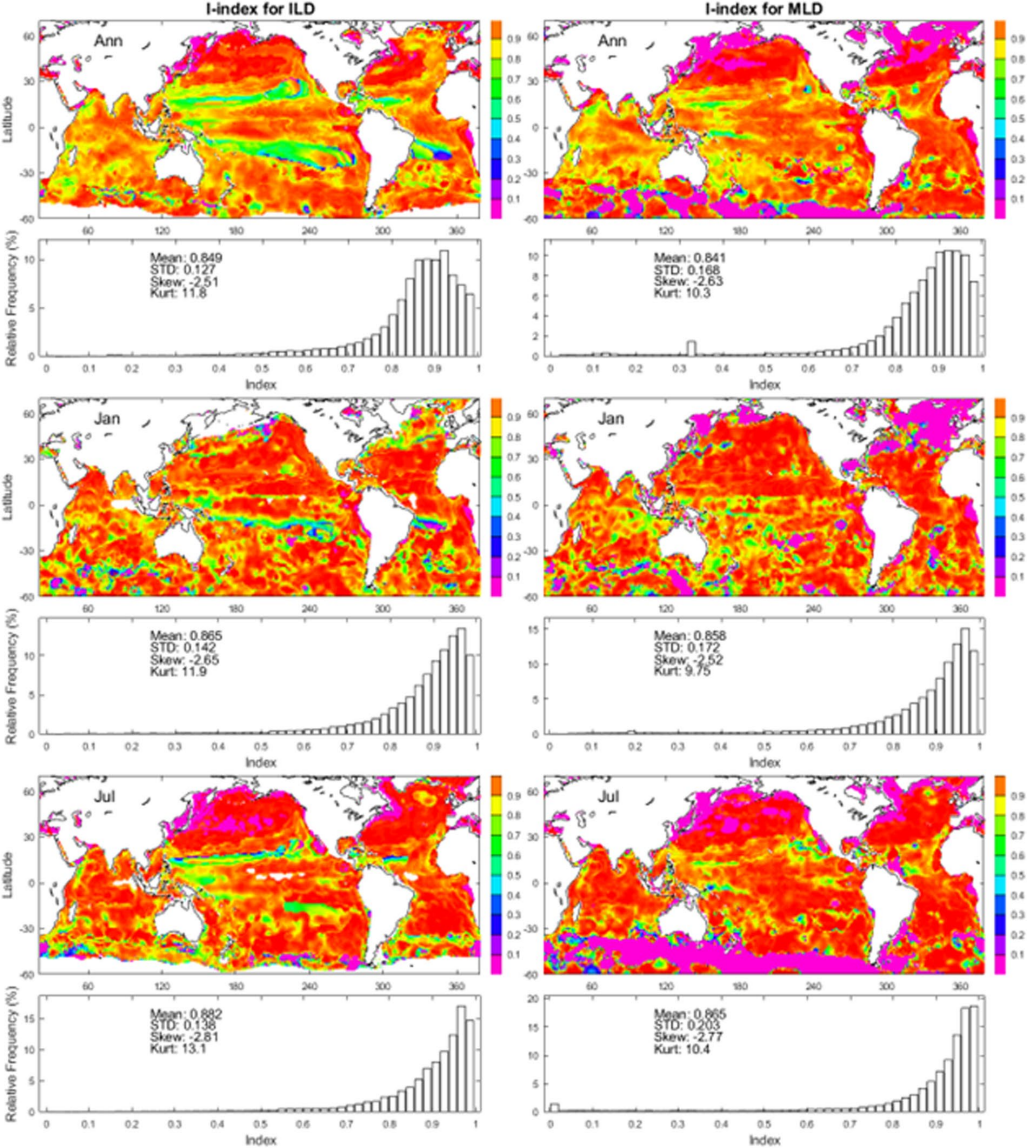
Maps and histograms of the *i*-index to determine (*h*_*T*_, *G*_*T*_) from the annual, January, July mean temperature profiles (left panels) and (*h*_*D*_, *G*_*D*_) from the annual, January, July mean density profiles (right panels). Here, top panels are for the annual mean; middle panels are for the January mean; and lower panels are for the July mean.

The annual, January, July mean maps and histograms of the i-index for ILD show high quality of the method. The global average i-index is 0.849 for the annual mean, 0.865 for the January mean, and 0.882 for the July mean (left panels in Fig. 13). The annual, January, July mean maps and histograms of the i-index for MLD show quality of the method. The global average i-index is 0.841 for the annual mean, 0.858 for the January mean, and 0.865 for the July mean (right panels in Fig. 13).

## Data Availability

The MATLAB codes to determine these thermohaline parameters from WOD18 (T, S) were published in the two related papers^28,29^, and can be obtained at 10.1007/s10872-017-0418-0. The main program is Thermohaline.m, along with four Matlab functions: validate.m, getgradient.m, ELGCore.m, and Iindex.m (see the Technical Validation Section). The function validate.m is employed to identify if (*T*, *ρ*) profiles having double gradient structure. The function ‘getgradient.m’ is used to calculate the vertical gradient. The function ELGCore.m is used to get (*h*_*T*_, *G*_*T*_) or (*h*_*D*_, *G*_*D*_) from T-profile or *ρ-*profile. The function Iindex.m is used to calculate the *i*-index (*I*_*ITL*_) for the error estimation. Then, the code ‘Thermohaline.m’ generates other thermohaline parameters such as ITL heat content, mixed layer fresh-water content, maximum thermocline gradient, thermocline depth, temperature at thermocline depth, maximum density gradient, pycnocline depth, density at pycnocline depth, barrier layer depth, and compensated layer depth. Since the calculation is local for individual (T, S) profile pair, interested readers may use our MATLAB codes to analyse any (T, S) profiles to get the derived thermohaline data with quality identification (i.e., *i*-index).
